# Association Between Initial Injection Volume and Radiographic Catheter Alignment in Continuous Deep Serratus Anterior Plane Block for Minimally Invasive Cardiac Surgery: A Single-Center Retrospective Observational Study

**DOI:** 10.7759/cureus.113569

**Published:** 2026-07-29

**Authors:** Ayano Toyama, Yusuke Iizuka, Kentaro Fukano, Taishi Saito, Takahiro Ueda

**Affiliations:** 1 Department of Anesthesiology and Critical Care Medicine, Jichi Medical University Saitama Medical Center, Saitama, JPN; 2 Department of Anesthesiology, Yokosuka City General Medical Center, Saitama, JPN

**Keywords:** catheter alignment, catheter insertion method, minimally invasive cardiac surgery, postoperative pain, serratus anterior plane block

## Abstract

Background

Continuous serratus anterior plane block (SAPB) is used for analgesia after thoracic and cardiac surgery. However, no standardized catheter placement method exists. This study investigated the association between initial injection volume and radiographic catheter alignment after minimally invasive cardiac surgery (MICS).

Methods

This retrospective cohort study included patients who underwent continuous SAPB after MICS between April 2021 and September 2024. Patients were divided into three groups based on initial bolus volume: low (≤ 30 mL), medium (40 mL), and high (≥ 50 mL). The primary outcome was radiographic catheter alignment assessed on frontal chest radiographs, categorized as favorable or unfavorable using predefined criteria. Secondary outcomes included fentanyl consumption and rescue analgesic administrations within 48 hours. Exploratory analysis evaluated associations between catheter alignment and analgesic use.

Results

The low-, medium-, and high-volume groups included 20, 33, and 45 patients, respectively. Favorable radiographic alignment was observed in 14 of 20 patients (70.0%) in the low-volume group, 32 of 33 patients (97.0%) in the medium-volume group, and 40 of 45 patients (88.9%) in the high-volume group (p = 0.015). Fentanyl consumption and rescue analgesic use did not differ significantly among groups. However, in an exploratory subgroup analysis, patients with favorable alignment required fewer rescue analgesic administrations than those with unfavorable alignment.

Conclusion

During continuous SAPB for MICS, an initial bolus volume of 40 mL or greater was associated with favorable radiographic alignment. The clinical relevance of these findings for postoperative analgesic outcomes warrants further prospective investigation.

## Introduction

Serratus anterior plane block (SAPB) is a fascial plane block in which local anesthetic is injected around the serratus anterior muscle to block the lateral cutaneous branches of the thoracic spinal nerves [1]. Compared with thoracic epidural anesthesia and thoracic paravertebral block, SAPB is performed in a more superficial fascial plane and may be advantageous in patients requiring postoperative anticoagulation because of its potentially lower risk of bleeding-related complications [2]. In minimally invasive cardiac surgery (MICS), continuous SAPB with catheter placement has been demonstrated to provide effective postoperative analgesia and facilitate early mobilization and recovery [3,4].

However, the initial local anesthetic injection volume used during catheter placement varies widely among studies [4-13], and its influence on catheter alignment remains unclear. Because both excessively large and excessively small injection volumes may have potential disadvantages, an appropriate balance in injection volume is likely important [14]. Larger injection volumes may facilitate hydrodissection of the fascial plane and improve catheter stability, but may also increase the risk of unintended catheter direction. Conversely, smaller injection volumes may result in suboptimal catheter positioning.

We aimed to evaluate the association between the initial injection volume during continuous SAPB catheter placement and postoperative radiographic catheter alignment after MICS, as well as postoperative analgesic consumption. We hypothesized that larger initial injection volumes would be associated with favorable radiographic catheter alignment and reduced postoperative rescue analgesic requirements.

## Materials and methods

Study design and setting

This single-center, retrospective cohort study was approved by the institutional review board of our institution (RIN S24-099). The study was conducted in accordance with the Declaration of Helsinki and was based on medical records collected between April 2021 and September 2024. The requirement for written informed consent was waived because this was a retrospective cohort study based on existing medical records. Instead, an opt-out approach was used, whereby patients were provided the opportunity to decline participation through information publicly posted on the department's website.

Participants

Patients who underwent MICS via right lateral thoracotomy and received continuous SAPB with catheter placement were included. Cases in which radiographic catheter alignment could not be assessed and cases in which the catheter was removed for medical reasons before radiographic assessment were excluded.　

Because this was a retrospective cohort study using an existing clinical dataset, all consecutive eligible patients available during the predefined study period were included to maximize the available sample size. Therefore, no a priori sample size calculation was performed.

Procedure for SAPB

First, after surgery, the patient was repositioned from the left semi-lateral decubitus position used during MICS to the full left lateral decubitus position for the block, with anesthesia maintained. Although this required a minor positional adjustment, it was performed without difficulty. A double-lumen endotracheal tube (35 Fr for most female patients and 37 Fr for most male patients) had been placed for the surgery, and one-lung ventilation using the left lung was applied during needle insertion to minimize the risk of pneumothorax in case of inadvertent pleural puncture.

Second, the injection site was determined using a linear-array ultrasound probe (L12-3, 3-12 MHz) with an ultrasound system (EPIQ 7G, Philips, Amsterdam, Netherlands) that was placed longitudinally along the mid-axillary line at the level of the fourth to sixth intercostal spaces to detect the serratus anterior muscle, a rib, and an intercostal muscle.

Third, after skin disinfection, a 17G Tuohy needle was inserted in a caudal-to-cranial direction using an in-plane technique. The needle tip was advanced to the plane between the serratus anterior muscle and the rib.

Fourth, after confirming negative aspiration, 0.25% levobupivacaine was injected (bolus volume: 10-60 mL, determined at the discretion of the attending anesthesiologist) to create a space between the deep surface of the serratus anterior muscle and the superficial layer of the intercostal muscle and rib. The syringe with the drug was connected to the needle via a tube, and an assistant other than the operator injected the drug under ultrasound guidance.

Fifth, an epidural catheter (Arrow FlexTip Plus, Teleflex, Wayne, PA, USA) was inserted through the needle and advanced 5 cm beyond the needle tip in the cranial direction. The single-lumen catheter with a single opening at the tip had a stainless-steel coil to prevent kinking and was covered with radiopaque polyurethane. Then, the catheter was secured to the skin with adhesive tape and, if necessary, sutured for additional stability.

Sixth, a patient-controlled analgesia pump (Coopdech Balloonjecter, Daiken Medical Co., Ltd., Osaka, Japan) was connected to the catheter (continuous infusion: 6 mL/h; bolus doses: 3 mL; lockout interval: 30 minutes) and filled with 300 mL of 0.25% levobupivacaine. The bolus dose and lockout interval were determined by the fixed specifications of the device. The continuous infusion rate can be set at 2, 4, or 6 mL/h, and the highest available rate (6 mL/h) was selected to ensure adequate analgesic delivery.

All punctures were performed by anesthesiology residents in postgraduate years three through six, under the direct supervision of board-certified anesthesiologists. A total of 25 residents participated during the study period.

Comparison groups

**Figure 1:**
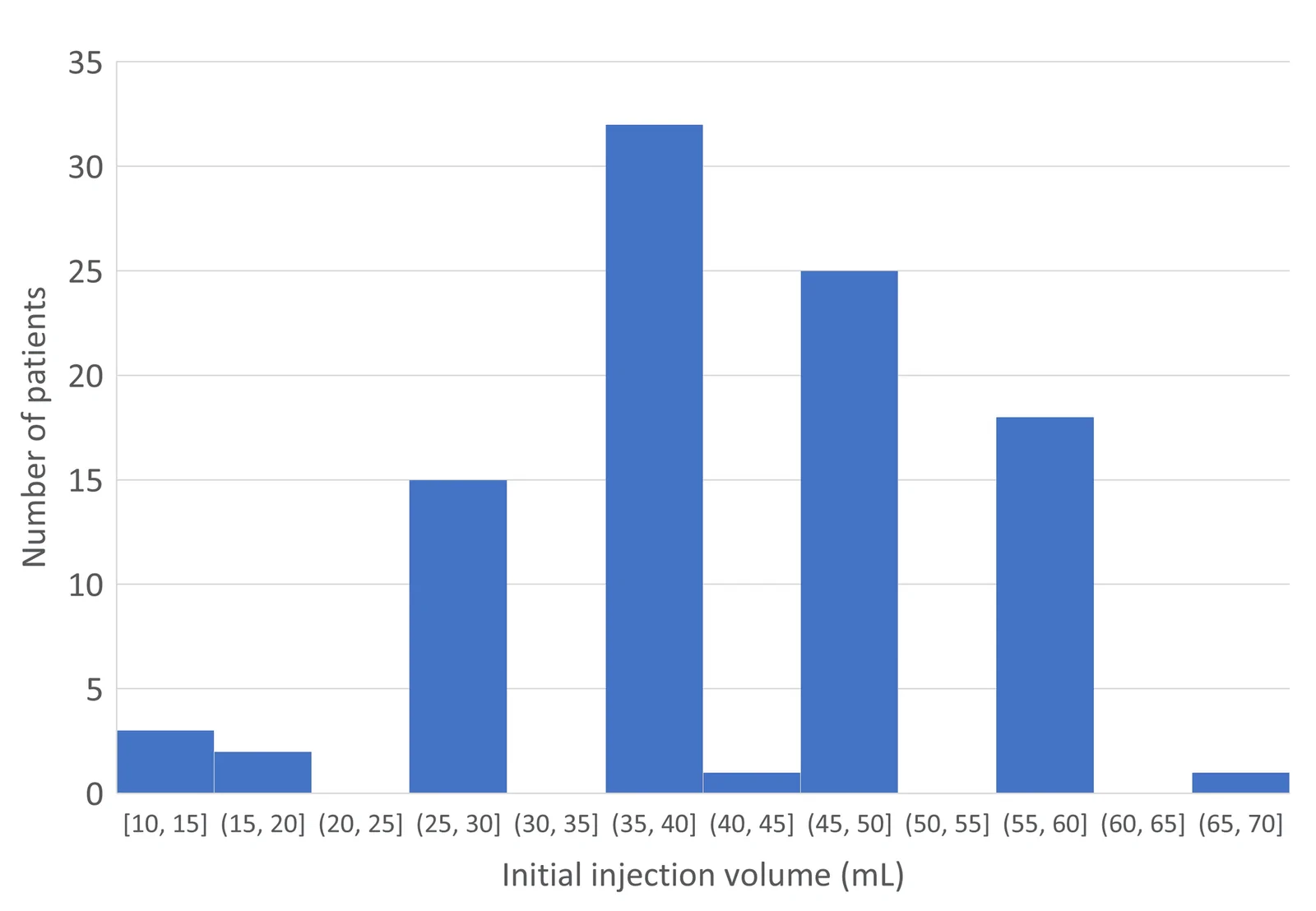
Distribution of initial injection volumes used before catheter placement during continuous serratus anterior plane block Histogram showing the distribution of initial injection volumes administered before catheter placement during continuous serratus anterior plane block. Patients were categorized into low-volume (≤30 mL), medium-volume (40 mL), and high-volume (≥50 mL) groups.

Patients were categorized into three groups based on the injection volume administered before catheter insertion: low-volume (≤ 30 mL), medium-volume (40 mL), and high-volume (≥ 50 mL). Injection volumes were not continuously distributed but clustered around 30, 40, and 50 mL in routine clinical practice during the study period (Figure 1). The low-volume group was based on typical single-shot doses used when SAPB was first introduced [1], commonly around 0.4 mL/kg (e.g., 20 mL for a 50 kg patient). The medium-volume group reflected the use of a moderately increased injection volume intended to create sufficient space for catheter insertion during continuous SAPB. The high-volume group was set near the maximum recommended dose for local anesthetics. The upper limit for levobupivacaine is 3 mg/kg, allowing up to 60 mL of 0.25% solution in a 50 kg patient.

Outcome measures

**Figure 2:**
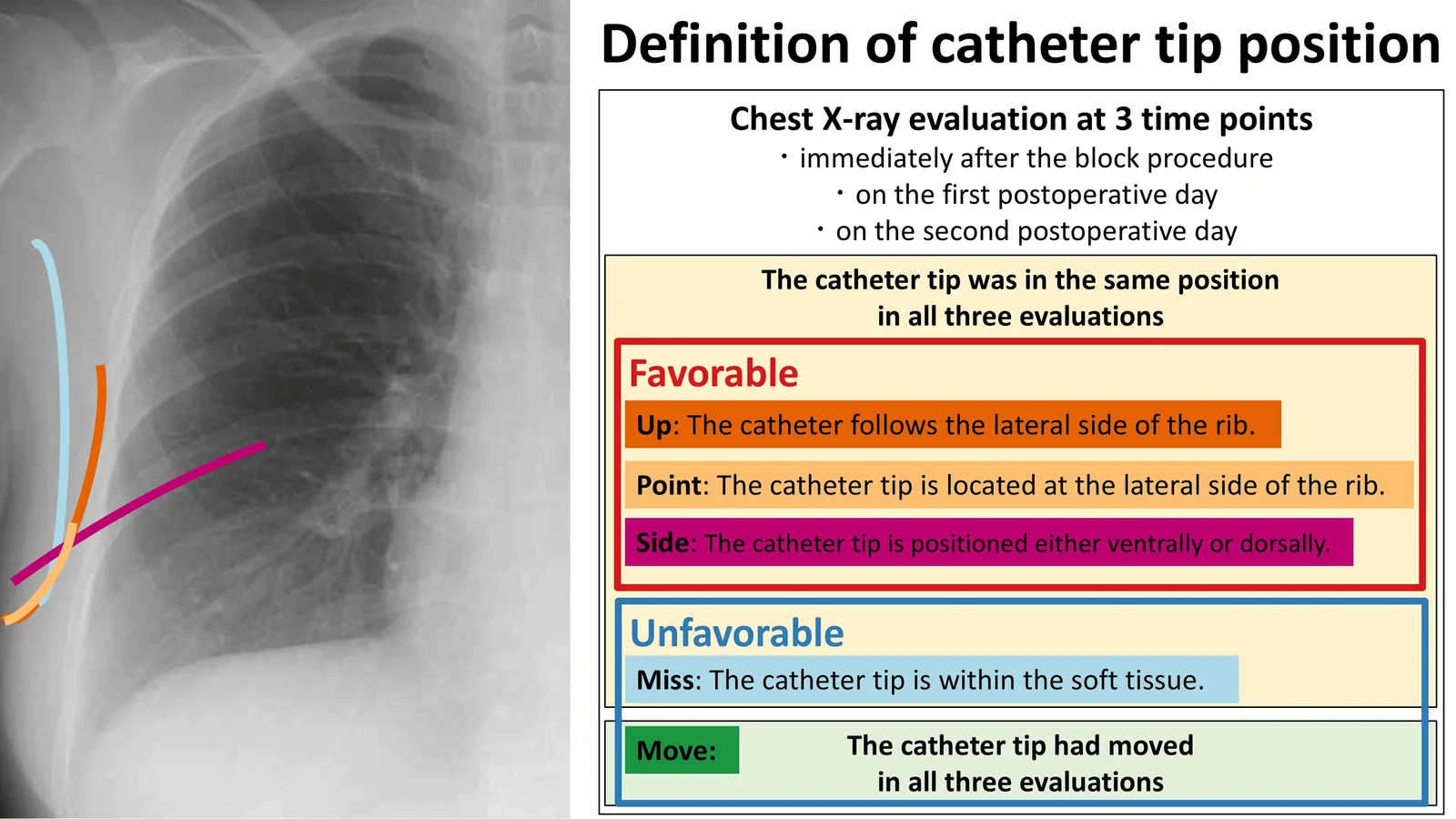
Evaluation of radiographic catheter alignment on chest radiographs Radiographic catheter alignment was assessed on serial chest radiographs obtained immediately after block placement and on postoperative days 1 and 2. “Up,” “Point,” and “Side” were classified as favorable alignment, whereas “Miss” and “Move” were classified as unfavorable alignment. This figure was manually created by the authors using Microsoft PowerPoint.

**Figure 3:**
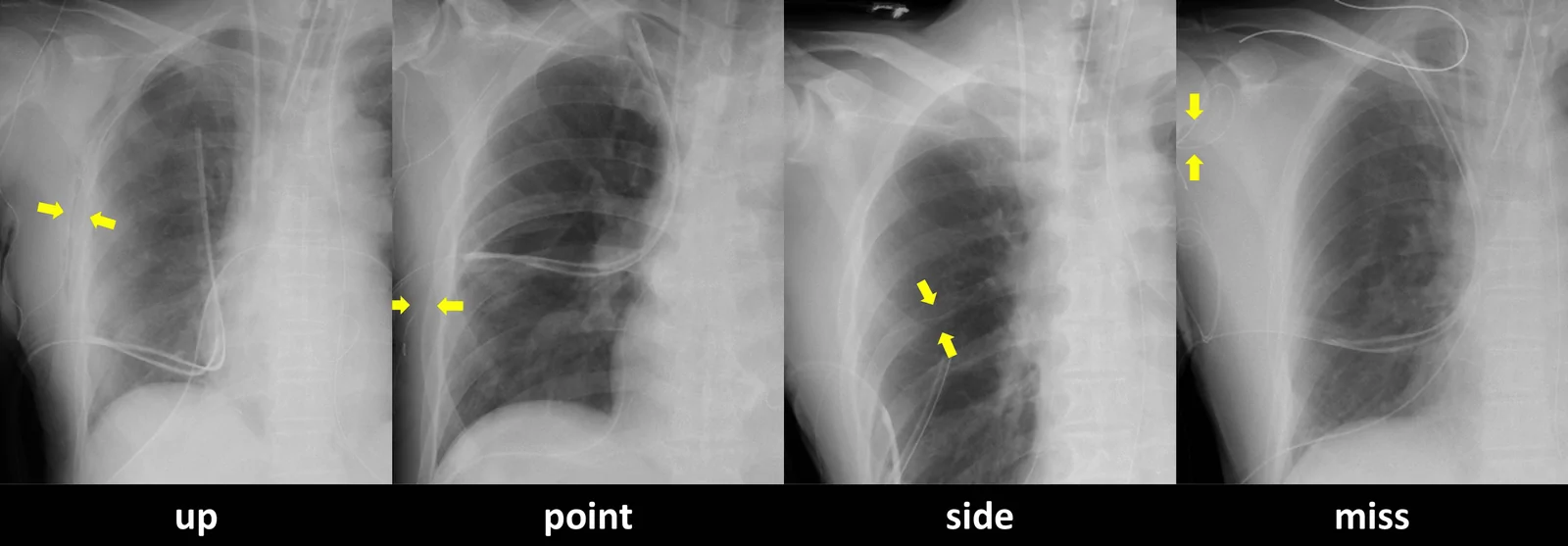
Representative postoperative chest radiographs of each catheter alignment category The catheter is indicated by the two yellow arrows. Catheter alignment was assessed using the outer contour of the rib as the anatomical landmark. The representative "up" image shows the catheter following the outer contour of the rib. In the "point" image, the catheter tip is projected at the outer edge of the rib. In the "side" image, the catheter tip is projected ventrally or dorsally relative to the rib. In the "miss" image, the catheter tip is projected within the soft tissue away from the rib.

The primary outcome was postoperative radiographic catheter alignment assessed using chest radiographs obtained at three time points. Radiographs were taken at three time points: immediately after the block procedure, on the first postoperative day, and on the second postoperative day. The catheter position was categorized as follows: “up” if the catheter followed the outer contour of the rib, “point” if the catheter tip was projected at the outer edge of the rib, “side” if the catheter tip was projected ventrally or dorsally, “miss” if the catheter tip was projected within soft tissue away from the rib, and “move” if catheter displacement was observed across the three radiographic assessments, including cases where the catheter dislodged spontaneously (Figures 2, 3).

Based on their radiographic relationship to the rib, “up,” “point,” and “side” were categorized as “favorable” because these patterns were considered more likely to reflect catheter positioning adjacent to the rib surface, where postoperative analgesic effects could be expected if the catheter was located within the appropriate serratus plane. In contrast, “miss” and “move” were categorized as “unfavorable” because these patterns were considered less likely to reflect appropriate catheter positioning. Chest radiographs were independently interpreted by an anesthesiologist. The reviewer was blinded to the clinical outcomes. Secondary outcomes were the cumulative fentanyl consumption within 48 hours after surgery and the number of rescue analgesic administrations. Rescue analgesics included intravenous acetaminophen (1,000 mg per dose, or 15 mg/kg for patients weighing ≤ 50 kg) and intravenous flurbiprofen axetil (50 mg per dose). The choice of postoperative analgesics was at the discretion of the attending Intensive Care Unit (ICU) physician and nurse.

Statistical analysis

Categorical variables were compared among the three groups using the chi-square test. Fisher’s exact test was used when expected cell counts were < 5. Because multiple comparisons were performed, Bonferroni correction was applied. Relative risks with 95% confidence intervals (CIs) for favorable radiographic catheter alignment were calculated using the low-volume group as the reference. Normality of continuous data was assessed using the Shapiro-Wilk test. Normally distributed data were presented as the mean ± standard deviation (SD) and analyzed using one-way analysis of variance when appropriate. Non-normally distributed data were presented as the median (interquartile range (IQR)) and analyzed using the Kruskal-Wallis test. An adjusted p value < 0.05 was considered statistically significant.

As an exploratory analysis, patients with an intubation time of 12 hours or less were analyzed to assess the association between radiographic catheter alignment categories and postoperative analgesic use. In this exploratory analysis, multivariable regression analyses were performed to evaluate the association between radiographic catheter alignment category and postoperative analgesic use, with adjustment for clinically relevant covariates. Based on clinical relevance, radiographic catheter alignment category, intubation time, and BMI were included as independent variables in the regression models. For count outcomes, generalized linear models with a log link were applied, and negative binomial regression was used when overdispersion was detected. For the analysis of fentanyl consumption within 48 hours, the dependent variable was log-transformed because of its skewed distribution, and β coefficients with 95% confidence intervals were calculated using multivariable linear regression. Statistical analyses were performed using R software (version 4.4.2; R Foundation for Statistical Computing, Vienna, Austria).

## Results

**Figure 4:**
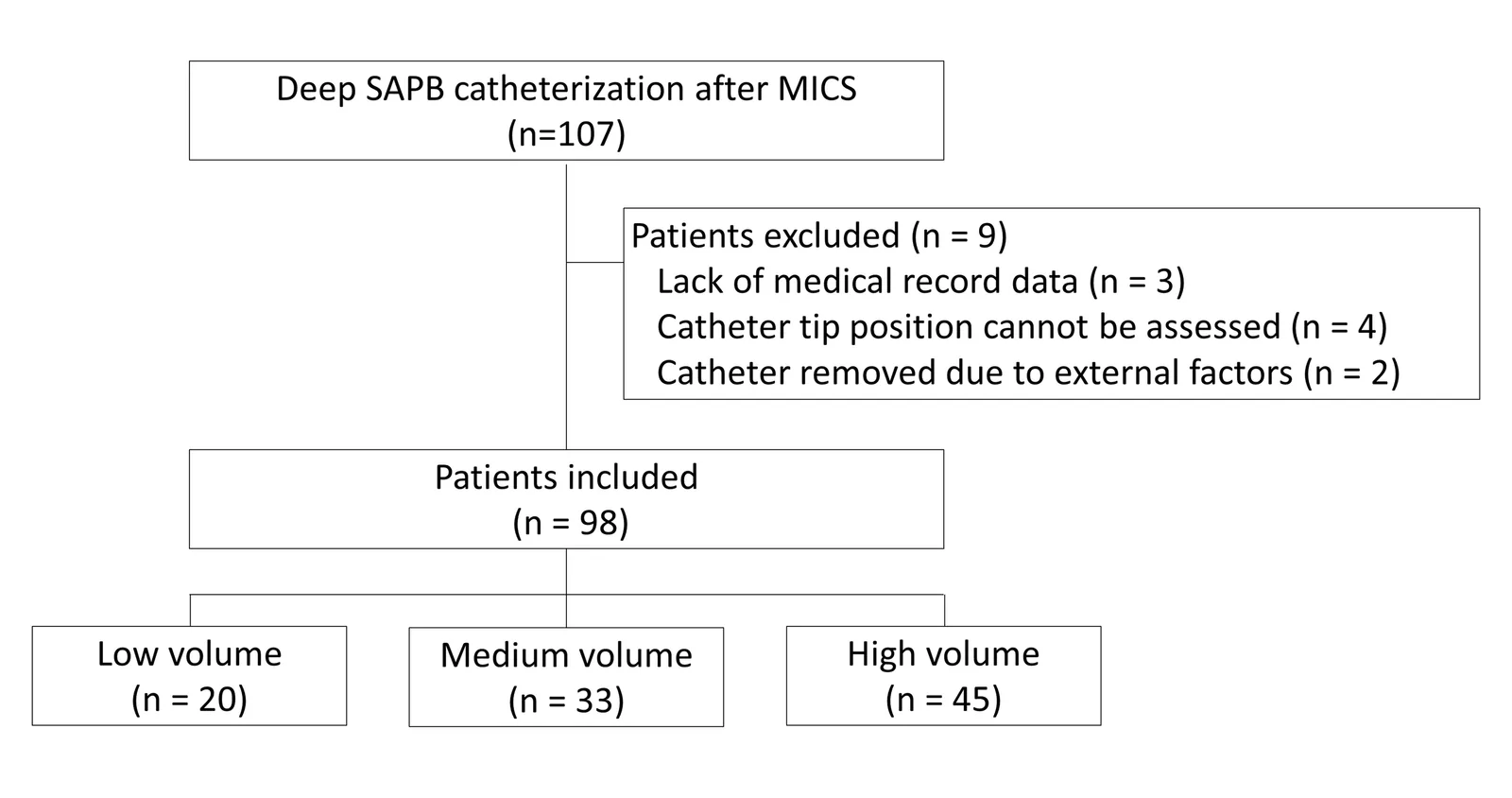
Flow chart of patient selection Among 107 patients who underwent deep serratus anterior plane block (SAPB) catheterization following minimally invasive cardiac surgery (MICS), 98 were included in the final analysis after exclusions. Patients were categorized according to the initial injection volume.

As shown in Figure 4, of the 107 patients initially enrolled in the study, nine were excluded, and 98 patients were included in the final analysis. Reasons for exclusion were the absence of medical record data (n = 3), inability to determine radiographic catheter alignment (n = 4), and catheter removal for reasons other than self-displacement (n = 2). The inability to adequately assess radiographic catheter alignment was primarily due to poor radiographic visualization associated with obesity or pulmonary edema. Among the two patients whose catheters were removed for non-self-displacement reasons, one underwent reoperation and the other experienced accidental catheter removal during chest drain adjustment.

Of the 98 patients included in the analysis, 20 were assigned to the low-volume group, 33 to the medium-volume group, and 45 to the high-volume group, based on the initial injection volume administered prior to catheter placement. Baseline demographic characteristics and surgical procedures did not differ significantly among groups (Table 1).

**Table 1:** Baseline demographic and perioperative characteristics according to initial injection volume group SD, standard deviation; BMI, body mass index; MICS, minimally invasive cardiac surgery; MVP, mitral valvuloplasty; AVR, aortic valve replacement; ASD, atrial septal defect; IQR, interquartile range.

Variable	Low volume (n=20)	Medium volume (n=33)	High volume (n=45)	p value
Male, n (%)	10 (50.0)	18 (54.5)	31 (68.9)	0.26
Age, years, mean ± SD	59.25 ± 14.72	61.06 ± 11.88	62.60 ± 12.19	0.61
Height, cm, mean ± SD	160.35 ± 13.08	161.42 ± 9.93	165.67 ± 8.78	0.078
Weight, kg, mean ± SD	57.70 ± 14.50	57.91 ± 9.24	62.62 ± 13.01	0.16
BMI, kg/m^2^,mean ± SD	22.19 ± 3.61	22.23 ± 3.03	22.70 ± 3.76	0.79
Surgical procedures, n (%)				
MICS-MVP	15 (75.0)	28 (84.8)	34 (75.6)	0.56
MICS-AVR	3 (15.0)	4 (12.1)	9 (20.0)	0.68
MICS-ASD closure	2 (10.0)	1 (3.0)	2 (4.4)	0.60
Intubation time, min, median (IQR)	603 (428-1061)	450 (425-75)	510 (418-90)	0.19

Primary outcome

Radiographic catheter alignment according to initial injection volume is summarized in Table 2. Detailed distributions of the five radiographic catheter position categories are shown in Table 3. The proportion of cases classified as “favorable” was 70.0% (14/20) in the low-volume group, 97.0% (32/33) in the medium-volume group, and 88.9% (40/45) in the high-volume group (p = 0.015). Multiple comparisons showed a significant difference between the low-volume and medium-volume groups (p = 0.026). Using the low-volume group as the reference, the relative risk of favorable radiographic catheter alignment was 1.39 (95% CI, 1.03-1.86) in the medium-volume group and 1.27 (95% CI, 0.94-1.72) in the high-volume group.

**Table 2:** Primary and secondary outcomes according to initial injection volume group *Bonferroni-adjusted pairwise comparisons for favorable radiographic alignment: Low vs medium, p = 0.026; low vs high, p = 0.23; medium vs high, p = 0.070. Relative risks (95% confidence intervals) for favorable radiographic alignment, using the low-volume group as the reference, were 1.39 (1.03–1.86) for the medium-volume group and 1.27 (0.94–1.72) for the high-volume group. IQR, interquartile range.

Variable	Low volume (n=20)	Medium volume (n=33)	High volume (n=45)	p value
Favorable radiographic alignment, n (%)	14 (70.0)	32 (97.0)	40 (88.9)	0.015^*^
Fentanyl dosage, μg, median (IQR)	221.00 (6.31-453.75)	171.00 (0.00-351.00)	143.00 (0.00-423.00)	0.59
Rescue analgesics, times, median (IQR)	1.00 (0.00-2.00)	0.00 (0.00-1.00)	1.00 (0.00-2.00)	0.27

**Table 3:** Radiographic catheter alignment for each initial injection volume *Bonferroni-adjusted pairwise comparisons for the “Up” category: Low vs medium, p = 0.018; low vs high, p = 0.42; medium vs high, p = 0.39. †Chi-square test. Up: χ²(2) = 9.29, Cramér's V = 0.31, p = 0.0096. Point: χ²(2) = 0.93, Cramér's V = 0.10, p = 0.63. ‡Fisher exact test.

Variable	Low volume (n=20)	Medium volume (n=33)	High volume (n=45)	p value
Up (%)	6 (30.0)	24 (72.7)	24 (53.3)	0.0095*^†^
Point (%)	7 (35.0)	8 (24.2)	11 (24.4)	0.62^†^
Side (%)	1 (5.0)	0 (0.0)	5 (11.1)	0.090^‡^
Miss (%)	3 (15.0)	1 (3.0)	2 (4.4)	0.21^‡^
Move (%)	3 (15.0)	0 (0.0)	3 (6.7)	0.067^‡^

Secondary outcomes

In the secondary analysis, neither cumulative fentanyl consumption nor the number of rescue analgesic administrations within 48 hours after surgery differed significantly among the three volume groups (Table 2).

In an exploratory analysis restricted to patients with an intubation time of 12 hours or less, baseline characteristics and surgical procedures were comparable between groups (Table 4). In an unadjusted comparison within this subgroup, the number of rescue analgesic administrations within 48 hours was lower in the favorable alignment group, with a median (IQR) of 0.00 (0.00-1.00) compared with 1.00 (1.00-1.75) in the unfavorable alignment group (p = 0.038) (Table 5). In multivariable negative binomial regression analyses adjusted for intubation time and BMI, favorable radiographic catheter alignment was independently associated with a lower number of rescue analgesic administrations within 48 hours (incidence rate ratio, 0.40; 95% CI, 0.19-0.88; p = 0.018) (Table 5). Similarly, radiographic catheter alignment category was not independently associated with cumulative fentanyl consumption within 48 hours in exploratory multivariable linear regression analyses using log-transformed data (Table 5). In contrast, BMI and intubation time were independently associated with cumulative fentanyl consumption within 48 hours. These analyses were exploratory and hypothesis-generating.

**Table 4:** Demographic Data Stratified by Radiographic Catheter Alignment Category (Patients With Intubation Time ≤12 h) SD, standard deviation; BMI, body mass index; MICS, minimally invasive cardiac surgery; MVP, mitral valvuloplasty; AVR, aortic valve replacement; ASD, atrial septal defect; IQR, interquartile range.

Variable	Favorable (n=57)	Unfavorable (n=6)	p value
Male, n (%)	31 (54.4)	3 (50.0)	1.00
Age, years, mean ± SD	60.19 ± 11.95	67.33 ± 13.06	0.17
Height, cm, mean ± SD	162.30 ± 10.83	159.17 ± 8.70	0.49
Weight, kg, mean ± SD	56.79 ± 12.22	55.17 ± 11.48	0.75
BMI, kg/m^2^,mean ± SD	21.47 ± 3.50	21.62 ± 3.12	0.91
Surgical procedures, n (%)			
MICS-MVP	45 (78.9)	4 (66.7)	0.60
MICS-AVR	10 (17.5)	2 (33.3)	0.32
MICS-ASD closure	2 (3.5)	2 (33.3)	1.00
Intubation time, min, median (IQR)	435.00 (395.00-490.00)	452.50 (397.50-511.25)	0.92

**Table 5:** Exploratory univariate and multivariable analyses of postoperative analgesic outcomes according to radiographic catheter alignment in patients extubated within 12 hours after surgery Univariate comparisons were performed between patients with favorable (n = 57) and unfavorable (n = 6) radiographic catheter alignment. Data are presented as median (interquartile range (IQR)) for the univariate comparisons. Incidence rate ratios (IRRs) and β coefficients with 95% confidence intervals (CIs) are presented for the multivariable analyses. Cumulative fentanyl consumption was log-transformed because of its skewed distribution. Negative binomial regression was used to account for overdispersion in the count data. BMI, body mass index.

Outcome	Variable	Estimate	95% CI	p value
Univariate comparison				
Fentanyl dosage, μg	Alignment (favorable vs unfavorable)	91.00 (0.00-205.00) vs 96.18 (2.84-301.00)		0.062
Rescue analgesics, times	Alignment (favorable vs unfavorable)	0.00 (0.00-1.00) vs 1.00 (1.00-1.75)		0.038
Multivariable analysis: Fentanyl dosage (Linear regression)				
	Alignment (favorable vs unfavorable)	β coefficient -0.314	-2.56 to 1.93	0.78
	Intubation time (per 1 min)	β coefficient 0.004	-0.012 to 0.004	0.34
	BMI (per 1 kg/m²)	β coefficient 0.25	0.056 to 0.44	0.012
Multivariable analysis: Rescue analgesics (Negative binomial regression)				
	Alignment (favorable vs unfavorable)	IRR 0.4	0.19 to 0.88	0.018
	Intubation time (per 1 min)	IRR 1	1 to 1	0.54
	BMI (per 1 kg/m²)	IRR 0.98	0.9 to 1.06	0.57

## Discussion

To the best of our knowledge, this is the first study to evaluate radiographic catheter alignment and postoperative analgesic outcomes according to the initial injection volume used during continuous SAPB. The initial injection volume varies across studies. SAPB was first described by Blanco et al., who reported an injection volume of 20-25 mL during the initial clinical application of this technique [1]. In previous reports, injection volumes administered prior to catheter insertion have ranged widely, including 3 mL [5-8], 10 mL [9], 15 mL [10], 20 mL [4], 25 mL [11], 30 mL [12], and 40 mL [13]. To the best of our knowledge, no studies have systematically evaluated how the initial injection volume influences radiographic catheter alignment.

This study demonstrated that an initial injection volume of ≥ 40 mL, administered prior to catheter insertion in continuous SAPB after MICS, was associated with a higher likelihood of favorable radiographic catheter alignment compared with injection volumes ≤ 30 mL. Although no clear improvement in postoperative analgesic outcomes was observed, this finding may still be relevant from a procedural perspective.

A higher proportion of cases classified as “favorable” was observed in the medium- and high-volume groups, primarily due to the increased frequency of radiographic catheter alignment categorized as “up” (Table 3). The needle was intentionally directed cranially during catheter placement to facilitate spread across multiple intercostal levels and toward the lateral cutaneous branches of the intercostal nerves. These findings suggest that an injection volume of approximately 40 mL or more may facilitate catheter advancement toward the intended target site. However, the high-volume group also exhibited a higher incidence of “side.” This finding suggests that excessive injection volume may cause the catheter to advance in an unintended direction even when catheter advancement appears technically successful. Importantly, this mechanism was not directly evaluated in the present study and should therefore be interpreted as a hypothesis. In contrast, radiographic catheter alignments classified as “unfavorable” occurred more frequently in the low-volume group. The initial injection volume used in this group was comparable to that reported in previous studies [4-12].

Despite the higher incidence of unfavorable radiographic catheter alignment in the low-volume group, postoperative analgesic outcomes did not differ significantly according to initial injection volume. These findings suggest that radiographic catheter alignment may not directly translate into clinically meaningful differences in analgesia. In exploratory analyses limited to patients extubated within 12 hours, favorable radiographic catheter alignment was associated with fewer rescue analgesic administrations but not with lower cumulative fentanyl consumption. Multivariable exploratory analyses likewise showed that radiographic catheter alignment was not independently associated with cumulative fentanyl consumption, whereas BMI and intubation time were independently associated with this outcome. These findings suggest that favorable catheter alignment may contribute to local analgesic efficacy, whereas overall postoperative opioid requirements are likely influenced by multiple patient- and perioperative factors beyond catheter position alone.

A speculative explanation for the lack of association between radiographic catheter alignment and postoperative analgesic outcomes is that radiographic catheter direction may not accurately reflect local anesthetic distribution relative to the surgical incision. Excessive cranial advancement (“up”) may cause local anesthetic spread above the target intercostal level, whereas lateral deviation (“side”) may place the catheter away from the lateral cutaneous branches of the intercostal nerves near the mid-axillary line [15]. However, because sensory block was not objectively assessed using a standard method such as pinprick testing [16], it remains unclear whether radiographic catheter orientation corresponded to the actual extent of sensory blockade. Therefore, these interpretations remain speculative.

Limitations

This study has several limitations.

First, frontal chest radiographs cannot determine the exact fascial plane location of the catheter. In the present study, because the serratus anterior muscle lies closely apposed to the rib surface, catheter alignment was estimated using the rib contour as an anatomical landmark. Although ultrasound provides detailed visualization of the fascial plane and surrounding anatomical structures [17], accurate catheter tip localization remains challenging, and no imaging modality has been universally adopted for routine postoperative assessment of perineural catheter position [18,19]. Nevertheless, chest radiography is routinely performed after cardiac surgery and provides a simple, noninvasive, and readily available method for postoperative catheter assessment.

Second, postoperative analgesic consumption may not fully reflect postoperative pain intensity. In our ICU, no standardized postoperative analgesic protocol was used. In addition, fentanyl consumption may have been influenced by endotracheal intubation because continuous fentanyl infusion was routinely administered to facilitate tolerance of endotracheal intubation. Furthermore, although oral analgesics, including tramadol-containing formulations, were prescribed after extubation, these medications were not incorporated into the analysis. Because oral analgesics varied in composition and dosing schedules, conversion to morphine milligram equivalents (MME) was difficult.

Third, postoperative pain is commonly evaluated using subjective assessment tools such as the numeric rating scale (NRS) [3,4,13]. However, NRS assessments were not routinely performed in our ICU during the study period, resulting in substantial missing data and precluding their inclusion in this retrospective analysis.

Fourth, there may have been variation in operator skill, catheter fixation methods, and other procedural factors during catheter placement. In addition, changes in operator experience over time may have influenced catheter alignment or stability. However, all blocks were performed under real-time ultrasound guidance by anesthesiology residents under the supervision of attending anesthesiologists, which likely ensured a reasonable degree of procedural consistency. Although block-related complications were not systematically assessed because they were not predefined study outcomes, no major block-related complications were documented in the medical records. Moreover, SAPB is generally considered a relatively simple fascial plane block compared with thoracic blocks performed on the posterior chest wall [20]. Therefore, the involvement of residents in needle placement may reasonably reflect real-world practice, as SAPB is commonly performed by trainees in clinical settings.

Finally, this was a retrospective single-center study in which all consecutive eligible patients during the predefined study period were included; therefore, no a priori sample size calculation was performed. The limited sample size may have reduced the statistical power to detect clinically relevant differences, particularly in postoperative analgesic outcomes, and may limit the generalizability of our findings. Further prospective multicenter studies, including randomized controlled trials where feasible, are warranted to validate these findings.

## Conclusions

This study demonstrated that an initial injection volume of ≥ 40 mL administered prior to catheter insertion for continuous SAPB after MICS was associated with more favorable radiographic catheter alignment compared with injection volumes ≤ 30 mL. Although initial injection volume itself was not clearly associated with postoperative analgesic outcomes, exploratory analyses suggested that favorable radiographic catheter alignment may be associated with reduced rescue analgesic requirements. Future prospective studies are needed to determine whether optimizing catheter alignment can improve not only the clinical effectiveness of continuous SAPB after MICS but also postoperative recovery within enhanced recovery pathways.
